# Progress in interventions for vaginal microecology

**DOI:** 10.3389/fcimb.2026.1888581

**Published:** 2026-08-19

**Authors:** Jie Li, Yang Liu, Tingting Gao, Hailing Ding, Rui Hu, Yunshan Wang, Bin Wu

**Affiliations:** 1 Department of Reproductive Medicine, Central Hospital Affiliated to Shandong First Medical University, Jinan, China; 2 Department of Clinical Laboratory, Xuanwu Hospital, National Clinical Research Center for Geriatric Diseases, Capital Medical University, Beijing, China; 3 College of Medicine, Qilu University of Technology, Jinan, China; 4 Jinan Central Hospital, Cheeloo College of Medicine, Shandong University, Jinan, China; 5 Basic Medical Research Center, Central Hospital Affiliated to Shandong First Medical University, Jinan, China

**Keywords:** combination therapy, hydrogel delivery, metagenomics, microecological intervention, probiotics, vaginal microbiome, vaginal microbiota transplantation

## Abstract

A balanced vaginal microbiome is fundamental to reproductive and gynecologic health, yet dysbiosis is common and clinically consequential. This narrative review synthesizes recent advances in microecological interventions, including probiotic, prebiotic, and synbiotic regimens; combination therapies; and vaginal microbiota transplantation. We place a particular focus on emerging delivery platforms like hydrogel-based carriers, which improve probiotic viability, mucosal adhesion, and controlled release. The review also explores how metagenomic analysis is refining community state typing, identifying pathogenic consortia, and enabling data-driven patient stratification and response monitoring. Despite these advances, key challenges remain, such as strain selection, functional validation, colonization durability, heterogeneous clinical endpoints, and clear regulatory pathways for live biotherapeutics. Future priorities must include developing functionally defined strain consortia, standardizing clinical outcomes, integrating multi-omics with biomaterials engineering, and conducting rigorous multicenter trials to deliver durable, safe, and truly individualized therapies.

## 1 Introduction

Vaginal dysbiosis is common and is associated with bacterial vaginosis, trichomoniasis, vulvovaginal candidiasis, sexually transmitted infections, and adverse reproductive outcomes (Brusselaers et al., 2019; Huang et al., 2024). Antibiotics remain first-line therapy for several vaginal infections, but their nonselective effects on resident microbiota and the high frequency of recurrence motivate investigation of microbiota-directed approaches (Moreno and Simon, 2019). This narrative review evaluates probiotics, prebiotics, synbiotics, combination regimens, biomaterial-based delivery, vaginal microbiota transplantation, and sequencing-guided precision strategies, with emphasis on the quality, limitations, and clinical relevance of the available evidence.

### 1.1 Search strategy

For this narrative review, PubMed/MEDLINE, Web of Science, Embase, and Scopus were searched from database inception to 30 June 2026. Search terms combined (vagina* OR cervicovaginal) AND (microbiota OR microbiome OR microecology OR dysbiosis) with (probiotic* OR prebiotic* OR synbiotic* OR *Lactobacillus* OR hydrogel OR nanotechnology OR vaginal microbiota transplantation OR VMT OR 16S rRNA OR shotgun metagenomic* OR long-read sequencing). English-language peer-reviewed human studies were prioritized; relevant animal and *in vitro* studies were included only when they addressed mechanism, biomaterial performance, or an emerging intervention without adequate clinical evidence. Reviews and consensus statements were used for definitions and to identify additional primary studies. Duplicate records, studies without direct relevance to vaginal microecology, non-peer-reviewed commercial claims, and reports lacking sufficient methodological detail were excluded. Titles and abstracts were screened for relevance, full texts were assessed narratively, and reference lists were hand-searched. Because this was a narrative rather than systematic review, study selection was not used to generate a pooled effect estimate.

## 2 Vaginal microecology

### 2.1 Composition of the vaginal microecology

In 1892, Albert Doderlein described non-spore-forming Gram-positive bacilli in vaginal secretions; these organisms were subsequently grouped within the genus *Lactobacillus* (Lamont et al., 2011; Kalia et al., 2020). Culture-based work by Walton et al. later confirmed that lactobacilli are frequently detected in healthy reproductive-age women (Walton, 1979). Modern molecular methods have since shown that vaginal communities are dynamic and that health-associated states are not defined solely by the presence of one genus.

Molecular approaches, including PCR and high-throughput sequencing, have expanded characterization of the vaginal microbiota (Srinivasan et al., 2010; Gajer et al., 2012). In 396 reproductive-age North American women, Ravel et al. defined five community state types (CSTs): CST I, II, III, and V were dominated by *L. crispatus*, *L. gasseri*, *L. iners*, and *L. jensenii*, respectively, whereas CST IV contained fewer lactobacilli and a more diverse assemblage of anaerobes, commonly including *Gardnerella*, *Prevotella*, *Dialister*, *Atopobium*, *Megasphaera*, *Sneathia*, *Peptoniphilus*, *Aerococcus*, and *Finegoldia* (Ravel et al., 2011). CST IV can be subdivided according to the relative abundance and composition of these taxa (Gajer et al., 2012).

To improve reproducibility across cohorts, France et al. developed VALENCIA (VAginaL community state typE Nearest CentroId clAssifier), a nearest-centroid method for assigning vaginal community state types (France et al., 2020). Subsequent validation and extension of reference centroids have facilitated comparison of 16S rRNA gene-sequencing datasets across studies (Fettweis et al., 2012). CST assignments are useful descriptive categories, but they should not be interpreted as universal disease states because community composition, symptoms, host factors, and functional activity may differ within a CST.

**Figure 1:**
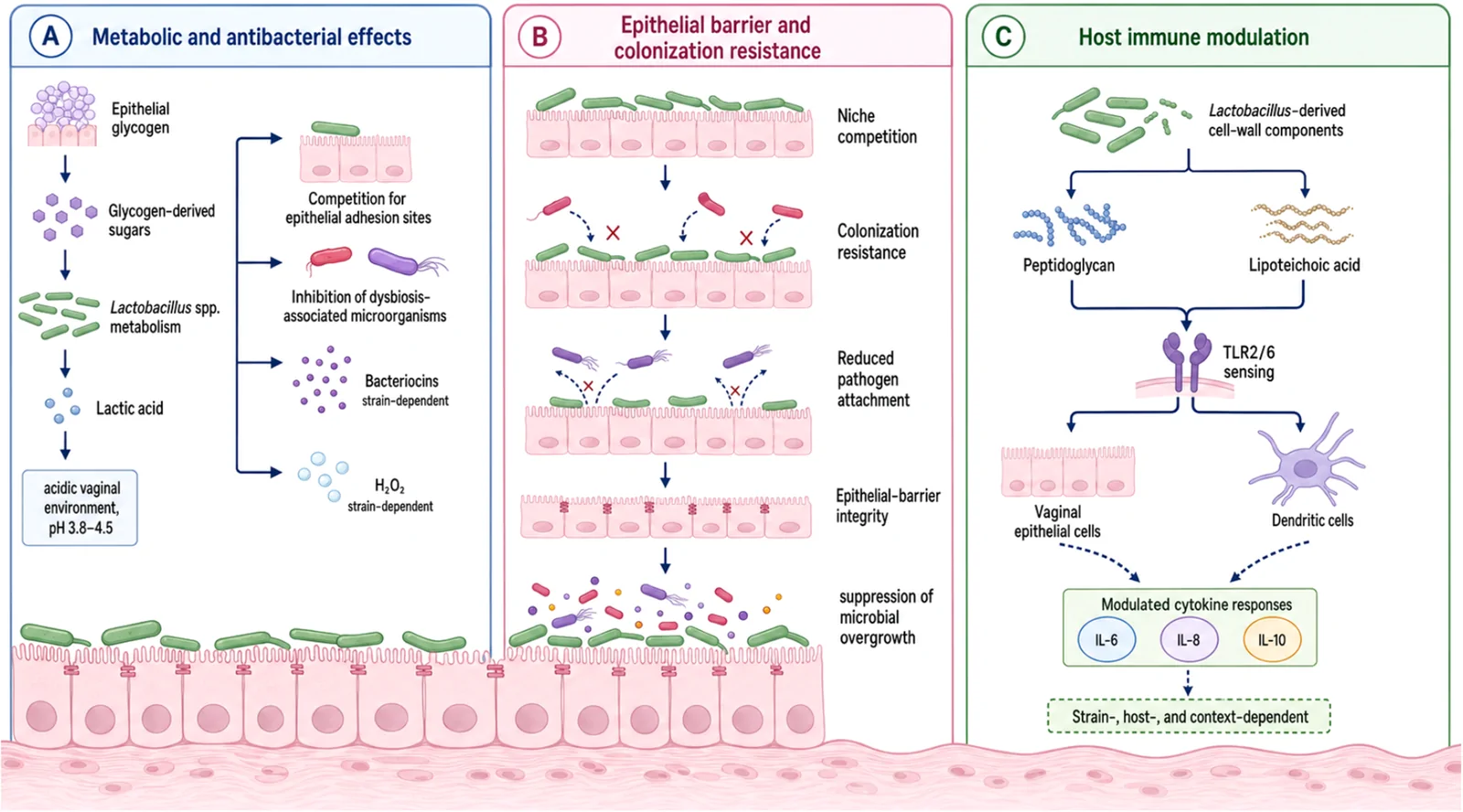
**(A)** Metabolic and antibacterial effects, including lactic acid production, competition for epithelial adhesion sites, and strain-dependent inhibition of dysbiosis-associated microorganisms. **(B)** Epithelial-barrier protection and colonization resistance through niche competition, reduced pathogen attachment, and suppression of microbial overgrowth. **(C)** Host immune modulation through Lactobacillus-derived cell-wall components and context-dependent cytokine responses.

Vaginal ecosystem stability reflects interactions among the microbiota, endocrine regulation, epithelial integrity, and local immunity (Smith and Ravel, 2017; Joseph et al., 2021). Health-associated lactobacilli contribute by producing lactic acid, maintaining a low pH, competing for epithelial adhesion sites, and, in a strain-dependent manner, producing bacteriocins or hydrogen peroxide (Hawes et al., 1996; Boskey et al., 2001; Juarez et al., 2003; Atassi and Servin, 2010; Stoyancheva et al., 2014; Petrova et al., 2015; Witkin and Linhares, 2017; Kovachev, 2018; Wang et al., 2021). These functions are not uniform across all *Lactobacillus* species or strains; therefore, genus-level abundance alone is insufficient to predict functional protection. The principal mechanisms are summarized in **Figure 1**.

### 2.2 The balance and imbalance of vaginal microecology

The vaginal microecological equilibrium denotes a consistently stable microbial population within the vagina, which is characterized by the following features: a) *Lactobacillus* dominance: the number of *Lactobacillus* in the vagina of healthy women accounted for 70% to 90% of the total bacterial community (Marnach et al., 2022). These microbes generate lactic acid, crucial for preserving the vaginal pH range of 3.8-4.5 and suppressing the proliferation of detrimental organisms; b) Low microbial diversity: a healthy vagina exhibits lower microbial diversity compared to other areas of the body, typically dominated by 1 to 3 species of *Lactobacillus* (Lee et al., 2020); c) Normal functioning of vaginal epithelial cells: these cells secrete antimicrobial substances and mucus, contributing to the maintenance of the microecological balance (Wira et al., 2005); d) Proper local immune function: the vagina’s local immune system can identify and eliminate pathogenic microorganisms, thereby helping to sustain the microecological balance (Mestecky and Fultz, 1999).

Vaginal dysbiosis is characterized by loss of health-associated lactobacilli, particularly *L. crispatus*, *L. gasseri*, and *L. jensenii*, together with increased abundance of anaerobic taxa such as *Gardnerella vaginalis*, *Atopobium vaginae*, *Prevotella* spp., *Megasphaera* spp., and *Sneathia* spp (MaChado and Cerca, 2015; Chee et al., 2020; Plummer et al., 2021). *L. iners* may persist during transitions between *Lactobacillus*-dominated and dysbiotic states and should not be treated as functionally equivalent to *L. crispatus*. Dysbiosis is commonly accompanied by higher vaginal pH, epithelial barrier disturbance, and altered local inflammatory signaling (Palacios et al., 2017; Lykke et al., 2021).

The composition of the vaginal microbiota varies with host genetics and ancestry-associated exposures, sexual and hygiene practices, hormonal state, age, menstruation, pregnancy, menopause, and antimicrobial or immunosuppressive therapy (Broome, 1989; Ravel et al., 2011; Hickey et al., 2012; MacPhee et al., 2013; Fettweis et al., 2014; Green et al., 2015; Lewis et al., 2017; Nonfoux et al., 2018; Zhang et al., 2018; Greenbaum et al., 2019; Gupta et al., 2020; Rasmussen et al., 2020; Schlievert, 2020; Chen et al., 2021; de Oliveira et al., 2021; Han et al., 2021; Sun et al., 2022). Population-level studies have reported differences in the prevalence of *Lactobacillus*-dominated communities, but these associations should not be interpreted as biologically deterministic or as evidence that one community structure is universally healthy. Health assessment should integrate symptoms, pH, microscopy or Nugent scoring, taxonomic composition, and relevant host factors.

**Figure 2:**
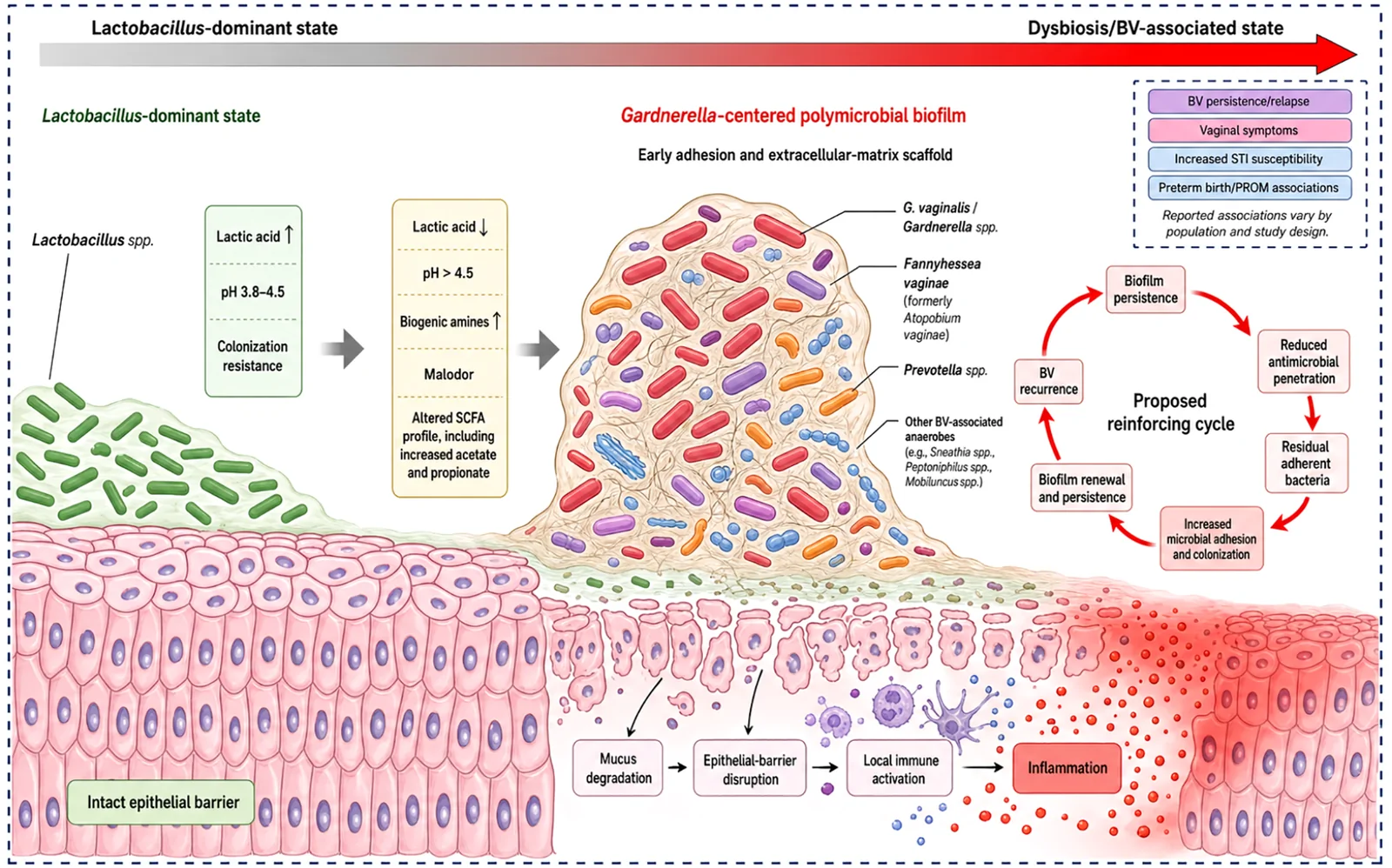
llustration contrasts a healthy vaginal Lactobacillus-dominant state with a dysbiotic, Gardnerella-centered polymicrobial biofilm. Shows transition to higher pH, reduced lactic acid, biofilm formation, epithelial disruption, local immune activation, inflammation, and a reinforcing cycle of biofilm persistence and BV recurrence, accompanied by clinical outcomes such as recurrent symptoms and increased infection susceptibility.

The Nugent score remains a widely used microscopy-based measure: scores of 0–3 are generally interpreted as *Lactobacillus*-predominant, 4–6 as intermediate, and 7–10 as consistent with bacterial vaginosis (Kusters et al., 2015). Vaginal dysbiosis is associated with bacterial vaginosis, vulvovaginal candidiasis, trichomoniasis, *Chlamydia trachomatis* infection, HIV acquisition, HPV persistence, adverse pregnancy outcomes, and reduced fertility (Petrin et al., 1998; Mastromarino et al., 2014; Johal et al., 2016; Santos et al., 2016; Filardo et al., 2017; Shannon et al., 2017; Amabebe and Anumba, 2018; Laniewski et al., 2018; Ranjit et al., 2018; Ceccarani et al., 2019; Gilbert et al., 2019; McKinnon et al., 2019; Kalia et al., 2020; Margarita et al., 2020; Fichorova et al., 2021; Glick et al., 2024; Wang et al., 2024). Most supporting studies are observational; therefore, these associations do not by themselves establish causality, and claims regarding HPV clearance, assisted reproduction, pregnancy, or infertility should be interpreted cautiously. The transition from a health-associated state to dysbiosis is illustrated in **Figure 2**.

## 3 Main strategies for vaginal microecological intervention

Microbiota-directed intervention aims to restore ecological function or re-establish a health-associated vaginal microbiota. The principal categories considered in this review are probiotics, prebiotics, synbiotics, combination regimens, experimental biomaterial-based delivery, and vaginal microbiota transplantation (O'Hanlon et al., 2013; Homayouni et al., 2014; Tachedjian et al., 2017; Li et al., 2019a). Their evidence is evaluated separately because route, biological mechanism, regulatory status, and clinical maturity differ substantially.

### 3.1 Framework for microbiota-directed interventions

Microbiota-directed interventions are best organized by biological role rather than by product label. Probiotics deliver viable, strain-defined microorganisms; prebiotics provide substrates selectively utilized by resident beneficial microorganisms; and synbiotics intentionally combine both components. Accordingly, *Lactobacillus* supplementation is treated as a probiotic strategy in Section 2.2.2.1 rather than as a separate evidence category. Evidence should be compared by route of administration, population, study design, sample size, endpoint, and follow-up duration (**Table 1**).

**Table 1:** Representative clinical studies of vaginal microecological interventions.

Study/reference	Design and sample	Intervention/comparator	Follow-up	Main endpoints and findings	Critical appraisal
De Alberti et al. (2015)	Randomized controlled pilot; n=40 healthy volunteers	Oral *L. acidophilus* La-14 + *L. rhamnosus* HN001 + bovine lactoferrin for 2 weeks	Days 7, 14, and 21	qPCR detection of administered strains increased at selected time points	Pilot in healthy participants; colonization was a surrogate endpoint and does not establish clinical efficacy
Mezzasalma et al. (2017)	Randomized, double-blind, three-arm pilot; n=60 (20/group)	Two oral multispecies probiotic mixtures versus placebo for 14 days	Days 7, 14, and 21	Increased vaginal detection of administered species by qPCR	Small pilot; short follow-up; detection and *in vitro* antagonism were not clinical disease endpoints
Hakimi et al. (2018)	Triple-blind randomized controlled trial; n=100 with BV	Prebiotic vaginal gel + oral metronidazole versus placebo gel + metronidazole for 7 days	Day 10 and 90 +/- 3 days	Higher cure rates by Amsel and Nugent criteria in the adjunct prebiotic group	Single-center study with a modest sample; replication and product-specific validation are needed
Heczko et al. (2015)	Multicenter randomized, double-blind, placebo-controlled trial; n=578 randomized for safety, n=241 microbiologically confirmed, n=154 completers for efficacy	Oral three-strain probiotic + standard/targeted antibiotics versus placebo + antibiotics	Initial 10-day course plus perimenstrual dosing for 3 months	Longer time to BV/AV relapse; lower vaginal pH and Nugent score; higher lactobacillus counts	Large randomized cohort, but substantial attrition and completer-based efficacy analysis may introduce bias
Laghi et al. (2014)	Multicenter double-blind randomized four-arm study; n=92 with BV	Three rifaximin vaginal regimens versus placebo	7 and 28 days after treatment	25 mg for 5 days produced the clearest short-term microbiome and metabolome shift toward a health-associated profile	Short follow-up; mechanistic microbiome/metabolome outcomes do not demonstrate durable recurrence prevention
DI Pierro et al. (2021)	Prospective open, uncontrolled pilot; n=35 HPV-positive women	Oral *L. crispatus* M247 for 90 days; no control group	90 days	CST shift toward *L. crispatus* dominance and reduced HPV positivity were observed	Small uncontrolled study; spontaneous HPV clearance and confounding prevent causal attribution
Lev-Sagie et al. (2019)	Exploratory case series; n=5 with intractable recurrent BV	Antibiotic pretreatment followed by one or more VMT procedures; no control group	5–21 months	Four of five achieved sustained clinical and microbiological remission; three required repeat VMT	Very small uncontrolled series; donor effects, repeat dosing, pathogen transmission, and long-term safety remain unresolved

AV, aerobic vaginitis; BV, bacterial vaginosis; CST, community state type; HPV, human papillomavirus; qPCR, quantitative polymerase chain reaction; VMT, vaginal microbiota transplantation.

### 3.2 Probiotics, prebiotics, and synbiotics

Microecological preparations encompass probiotics, prebiotics, and synbiotics (Shenderov, 2013; Zhang et al., 2018; Ondrussek-Sekac et al., 2021). Probiotics are live microorganisms administered at an adequate dose, prebiotics are substrates selectively utilized by host microorganisms, and synbiotics combine microorganisms with a substrate intended to support their function. Evidence derived from the intestinal microbiota should not be assumed to apply to the vagina because the ecological niches, dominant taxa, exposure routes, and clinical endpoints differ.

#### 3.2.1 Mechanism of action of microecological agents

These interventions may act through complementary mechanisms: increasing the abundance or activity of health-associated lactobacilli, lowering vaginal pH through lactic-acid production, competing with pathogens for nutrients and adhesion sites, producing antimicrobial metabolites, and modulating epithelial and mucosal immune responses (Menard et al., 2004; Li et al., 2019a; Monteagudo-Mera et al., 2019; Artym and Zimecki, 2021; Cunningham et al., 2021; Liu et al., 2021; Nicolo et al., 2021; Croatti et al., 2022; Gholiof et al., 2022; Lin et al., 2024; Supriya et al., 2024; Li et al., 2019). The magnitude of each effect is strain-, substrate-, dose-, route-, and host-dependent; mechanistic plausibility should therefore be distinguished from demonstrated clinical benefit.

#### 3.2.2 Classification of microecological preparations

##### 3.2.2.1 Probiotics

Probiotics are defined as live microorganisms that, when administered in adequate amounts, confer a health benefit on the host (Lilly and Stillwell, 1965; Hill et al., 2014; Jager et al., 2019). Vaginal applications have focused mainly on strain-defined lactobacilli and, less frequently, bifidobacteria. Taxonomic identity at the genus or species level is not sufficient to infer efficacy: acid production, epithelial adhesion, antimicrobial activity, safety, and colonization durability can differ substantially among strains (Han and Ren, 2021).

Probiotic effects are expected to be strain-specific. Candidate vaginal lactobacilli may transiently adhere to mucosa, compete with pathogens, generate lactic acid or bacteriocins, and influence epithelial or immune signaling (Palm et al., 2015; Metzger et al., 2018; Mei and Li, 2022). However, detection of an administered strain does not necessarily demonstrate durable engraftment, symptom improvement, or prevention of recurrence; these outcomes require controlled clinical assessment.

Clinical evidence for probiotics is heterogeneous. Two small randomized pilot studies in healthy women (n=40 and n=60) primarily evaluated short-term vaginal detection of orally administered strains rather than treatment of disease (De Alberti et al., 2015; Mezzasalma et al., 2017; Sekito et al., 2023; Atkins et al., 2024). In contrast, trials in bacterial vaginosis have used clinical cure, Nugent score, vaginal pH, recurrence, or microbiota composition as endpoints, with variable formulations and follow-up (Brotman, 2011; Donders et al., 2011; Heczko et al., 2015; Abruzzo et al., 2018; Ma et al., 2022; Ang et al., 2023). These differences, together with strain specificity and incomplete reporting of colonization, limit direct comparison and preclude class-wide efficacy claims. Evidence from non-vaginal settings likewise cannot be directly extrapolated to vaginal indications (Chang et al., 2017).

##### 3.2.2.2 Prebiotics

The International Scientific Association for Probiotics and Prebiotics defines a prebiotic as a substrate that is selectively utilized by host microorganisms and confers a health benefit (Ng et al., 2019). Candidate vaginal prebiotics include selected oligosaccharides and other compounds intended to favor health-associated lactobacilli (Kazmierczyk-Winciorek et al., 2021; Saxami et al., 2023; Xie et al., 2023; Guarner et al., 2024). A compound should not be labeled a vaginal prebiotic solely because it is a dietary fiber or supports bacterial growth *in vitro*; selective utilization and a host benefit should be demonstrated in the relevant vaginal context.

Proposed vaginal prebiotic mechanisms include selective support of lactobacilli, enhancement of mucosal adhesion, increased lactic-acid production, suppression of pathogen growth, and modulation of inflammatory signaling (Andersen et al., 2011; Delgado-Diaz et al., 2019; Cai et al., 2020; Armstrong and Kaul, 2021; Rousseaux et al., 2023; Cai et al., 2024; Du et al., 2024; Matsuzaki et al., 2024). Much of this evidence is mechanistic or preclinical, and effects cannot be generalized across substrates. Studies should report the tested compound, concentration, route, comparator, microbiological method, and clinically meaningful outcomes.

Clinical evidence for vaginal prebiotics remains limited. In a triple-blind randomized trial of 100 women with bacterial vaginosis, a 7-day prebiotic vaginal gel plus oral metronidazole improved Amsel- and Nugent-defined cure at days 10 and 90 compared with placebo gel plus metronidazole (Hakimi et al., 2018; Pino et al., 2022). A systematic review found possible benefit for pro/prebiotics alone or with antibiotics but emphasized heterogeneity in formulations, comparators, and recurrence definitions (Afifirad et al., 2022). These findings support further product-specific trials rather than a general recommendation for all prebiotics.

##### 3.2.2.3 Synbiotics

Synbiotics combine live microorganisms with a substrate selectively utilized by the co-administered microorganisms or by resident microbiota (Young, 1998; Wan et al., 2019; Pandey et al., 2015). For vaginal use, the rationale is to pair a strain-defined probiotic with a compatible substrate that supports survival or metabolic activity in the vaginal niche. A mixture should not be described as synergistic unless the combined effect is shown to exceed that of its individual components.

Potential synbiotic effects include enhanced survival of administered lactobacilli, increased lactic-acid production, competitive exclusion of pathogens, and modulation of epithelial or immune responses (Kaplan and Hutkins, 2003; Gibson et al., 2017; Markowiak and Slizewska, 2017; Zuniga et al., 2018; Ji et al., 2023; Maftei et al., 2024). These mechanisms overlap with those of probiotics and prebiotics and are therefore summarized here rather than repeated as independent class-wide effects. Demonstration of compatibility, selective substrate use, and added benefit from the combination is essential.

Evidence for vaginal synbiotics is less mature than the wording of many reports suggests. Available studies vary in formulation, route, diagnostic criteria, and follow-up, and some mechanistic support derives from animal models rather than human trials (Ling et al., 2013; Chee et al., 2020; Afifirad et al., 2022; Savicheva et al., 2023; Toquet et al., 2025). Animal findings can inform biological plausibility but cannot establish human efficacy or safety. Adequately powered randomized trials should compare the combination with each component and with standard therapy.

#### 3.2.3 Recent research progress on microecological agents

##### 3.2.3.1 Diversification and precision of probiotic preparations

Species and strain differences are central to probiotic development. *L. crispatus* is frequently associated with low-diversity, lactic-acid-producing communities, whereas *L. iners* has a smaller genome and is often detected during transitions between community states (Ravel et al., 2011; Walther-Antonio et al., 2014; France et al., 2016). These associations do not guarantee therapeutic performance. Candidate strains, including those explored for HPV-associated outcomes, require functional characterization and controlled clinical validation before disease-specific claims can be made (Brussow, 2019; Chen et al., 2023).

##### 3.2.3.2 Combination of nanotechnology and microecological preparations

Nanoparticles and other engineered carriers may protect probiotics, modify release kinetics, or improve mucosal retention (Centurion et al., 2021; Dangi et al., 2023; Peng et al., 2023; Senthil Kumar and Sheik Mohideen, 2024; Wei et al., 2024; Zhu et al., 2024). At present, most supporting evidence comes from *in vitro* experiments, material-characterization studies, or animal models. These platforms should therefore be described as preclinical delivery concepts rather than established vaginal therapies. Translation requires toxicology, biodistribution, mucosal-compatibility, dose, manufacturing, and long-term safety data in addition to evidence of microbiological and clinical benefit.

#### 3.2.4 Application cases of microecological preparations in restoring the microecological balance of the vagina

Representative studies illustrate the heterogeneity of the evidence (**Table 1**). Antibiotic-probiotic trials for bacterial vaginosis have reported improvements in recurrence-related or microbiological outcomes, but formulations and analysis populations differ (Heczko et al., 2015; Vasundhara et al., 2021). A randomized four-arm rifaximin study (n=92) identified short-term microbiome and metabolome changes, with the 25-mg 5-day regimen performing best among the tested doses, but follow-up was limited to 28 days (Laghi et al., 2014). An open, uncontrolled study of 35 HPV-positive women reported CST shifts and reduced HPV positivity after 90 days of oral *L. crispatus* M247 (DI Pierro et al., 2021; Palma et al., 2018); because there was no control group, spontaneous viral clearance and confounding preclude attribution of HPV clearance to the probiotic. Observational associations with endometrial repair, pregnancy, infertility, or assisted reproduction likewise should not be interpreted as proof that probiotics improve reproductive outcomes (Wronding et al., 2023).

Overall, the clinical evidence is limited by small or selectively analyzed samples, short and inconsistent follow-up, strain and product heterogeneity, variable comparators, and nonstandardized endpoints (**Table 1**). For example, the multicenter trial by Heczko et al. randomized 578 participants for safety, but only 241 had microbiologically confirmed BV/AV and 154 completers contributed to the primary efficacy analysis, creating potential attrition and per-protocol bias (Heczko et al., 2015). Future trials should prespecify sample-size calculations, intention-to-treat analyses, strain-level identification, adherence, adverse events, durable recurrence, and patient-centered outcomes.

### 3.3 Combination therapy

Combination regimens most commonly pair antibiotics with probiotics or prebiotics. A multicenter randomized trial suggested that adjunct oral probiotics prolonged time to BV/AV relapse after antibiotic therapy, but attrition and completer-based analysis temper confidence (Heczko et al., 2015; Wang et al., 2019). Evidence linking vaginal microbiota to IVF or pregnancy outcomes is largely observational, and studies of probiotic use in pregnancy vary in population and endpoint (Vitali et al., 2012; Mitra et al., 2016; Taylor et al., 2017; Haahr et al., 2019; Fernandez et al., 2024). Accordingly, combination therapy may improve selected microbiological or BV-related outcomes, but current evidence does not establish improved ART success, prevention of preterm birth, or treatment of infertility. Safety assessment is especially important in pregnancy and immunocompromised populations.

The integration of diverse microecological intervention strategies has demonstrated significant benefits for the restoration of vaginal microecological equilibrium, thereby representing a promising direction for therapeutic development. Nevertheless, the optimization of combination therapy strategies continues to face several challenges. First, the timing and interval of probiotic administration relative to antibiotics remain undefined. Concurrent administration may result in direct eradication of probiotics by antibiotics, thereby diminishing efficacy, whereas the optimal time window for sequential administration (i.e., supplementation of probiotics after completion of antibiotic therapy) has yet to be established. Second, the compatibility between antibiotics and probiotic strains is critical, as some broad-spectrum antibiotics may also exhibit bactericidal activity against the *Lactobacillus* strains intended for intervention. Thus, targeted selection of strains resistant to specific antibiotics is required. Finally, the long-term effects of combination therapy on the microbiome, including the potential unintended promotion of antibiotic resistance gene dissemination within microbial communities, warrant further investigation and surveillance. These issues underscore the necessity of conducting more refined mechanistic studies and protocol optimization before combination therapies can be widely adopted in clinical practice.

### 3.4 Hydrogel carrier delivery

Hydrogels can encapsulate live microorganisms or other active agents and may improve local retention, protect viability, and permit controlled release (Koivusalo et al., 2019; Patarroyo et al., 2021; Abdullah et al., 2022; Chen et al., 2023; Deshkar et al., 2023; Yu et al., 2024; Zhao et al., 2025; Xiao et al., 2020; Qiu et al., 2023; Zhao et al., 2023). These properties are formulation-dependent and should be demonstrated under physiologically relevant vaginal conditions, including acidic pH, mucus, mechanical stress, and interactions with resident microbiota. Material performance alone is not evidence of clinical efficacy.

Evidence for probiotic-hydrogel and nano-enabled vaginal delivery is predominantly preclinical. The FeLab hyaluronic-acid hydrogel containing rGO@FeS2 nanozymes and lactobacilli was evaluated through laboratory and animal experiments for *Candida albicans* vaginitis (Wei et al., 2023); it should not be presented as an established clinical treatment. A cellulose-based hydrogel study demonstrated encapsulation and controlled release of *L. paracasei* outside a vaginal clinical setting (Li et al., 2023), and the PrePOP publication is a randomized-trial protocol rather than an efficacy result (Corbett et al., 2025). Thus, claims of improved pregnancy outcomes, low recurrence, or clinical safety cannot yet be supported by these studies.

Hydrogel-based delivery remains a promising research direction, but clinical translation requires staged evaluation. Priorities include reproducible manufacturing, viability during storage, release under vaginal conditions, local and systemic toxicology, effects on epithelial integrity and resident microbiota, and adequately powered randomized trials with durable clinical endpoints. Until such evidence is available, hydrogels and nanomaterials should be described as experimental platforms rather than technologies likely to revolutionize near-term clinical care.

### 3.5 Vaginal microbiota transplantation

Vaginal microbiota transplantation (VMT) transfers a donor vaginal microbial community to a recipient with the aim of restoring a health-associated ecosystem. The approach is experimental and has been studied mainly in refractory recurrent bacterial vaginosis (Martinelli et al., 2023; Jawanda et al., 2024; Luo et al., 2024). It differs from administration of a defined probiotic because the inoculum contains a complex, incompletely characterized microbial community and potentially transferable nonbacterial components.

Clinical evidence is limited to small exploratory reports. In the first case series, five women with intractable recurrent bacterial vaginosis received VMT; four achieved clinical and microbiological remission during 5–21 months of follow-up, but three required repeat transplantation and one required a donor change (Lev-Sagie et al., 2019). The absence of a control group, the very small sample, variable dosing, and concomitant antibiotic pretreatment prevent conclusions about efficacy, optimal protocol, or uncommon harms.

Safety and regulation are central barriers to VMT. Donor screening should address sexually transmitted infections, blood-borne viruses, bacterial and fungal pathogens, antimicrobial-resistant organisms and resistance genes, recent antibiotic exposure, pregnancy, immunologic and metabolic conditions, and behaviors that alter transmission risk. Standardized collection, processing, storage, dose, route, traceability, biobanking, and recipient follow-up are needed. Residual risks include undetected or emerging pathogens, transfer of undesirable functions, donor-dependent engraftment, immune effects, and late adverse events. Ethical issues include informed consent for uncertain long-term risks, donor privacy, compensation, reproductive implications, and management of incidental findings. VMT should therefore remain within ethically approved, prospectively registered clinical studies under an appropriate regulatory framework.

### 3.6 Barriers to clinical translation and commercialization

Although microecological interventions have shown great promise in theory and in early clinical studies, their successful translation from laboratory to market faces multiple obstacles.

The first is regulatory complexity. Live biotherapeutic products (LBPs), such as probiotics and VMT formulations, currently face an uncertain regulatory pathway worldwide. Regulatory agencies, including the U.S. Food and Drug Administration (FDA) and the European Medicines Agency (EMA), require rigorous data on safety, efficacy, and manufacturing consistency, leading to high development costs and prolonged timelines.

Second, chemistry, manufacturing, and controls (CMC) pose substantial technical challenges. Ensuring the viability, purity, and batch-to-batch consistency of live biotherapeutic preparations during production, transport, and storage is particularly difficult. Establishing stable, scalable production processes and reliable quality control standards remains a key bottleneck for commercialization.

Finally, the design and execution of clinical trials remain highly challenging. Vaginal microbiota is dynamic and subject to significant inter-individual variability, and placebo effects are often pronounced in related trials. These factors complicate trial design, patient recruitment, and endpoint evaluation. Moreover, translating high-dimensional data from metagenomics into clinically actionable biomarkers for patient stratification and efficacy assessment remains an unresolved issue. Overcoming these obstacles will require close collaboration among academia, industry, and regulatory authorities.

## 4 Development of metagenomics and individualized vaginal microecological interventions

Sequencing approaches should be distinguished clearly. 16S rRNA gene amplicon sequencing profiles bacterial community composition at limited taxonomic resolution and does not directly measure genes or pathways. Shotgun metagenomic sequencing analyzes total community DNA and can provide strain-level, functional-gene, and antimicrobial-resistance information when sequencing depth and reference databases are adequate. Long-read sequencing can improve assembly and strain resolution but has distinct error, cost, and analytical requirements (Fierer et al., 2012; Bharti and Grimm, 2021; Singleton et al., 2021; Lugli et al., 2023; Olivier et al., 2023; Kang et al., 2024). The term metagenomics is used here for shotgun community-DNA analysis; 16S rRNA profiling is described separately and is not termed ‘shotgun metagenomics’ or ‘metagenomics.’

Shotgun metagenomics can characterize taxonomic composition and functional potential, including depletion of health-associated taxa, pathogenic consortia, metabolic pathways, and resistance genes. These data may support patient stratification or selection of targeted interventions (Goltsman et al., 2018; Holm et al., 2023; Hasan et al., 2024; Biagioli et al., 2025; You et al., 2019). However, functional potential inferred from DNA is not equivalent to gene expression or metabolite activity, and treatment recommendations based solely on sequencing remain investigational. Integration with symptoms, microscopy, pH, host factors, and validated clinical endpoints is necessary.

Major limitations include variation in sampling site and timing, DNA extraction, sequencing platform, reference database, bioinformatic pipeline, contamination control, and taxonomic nomenclature. 16S rRNA gene sequencing may not reliably resolve closely related lactobacilli, whereas shotgun and long-read methods require greater cost, depth, and analytical expertise. Cross-sectional associations cannot establish causality, and externally validated thresholds for clinical decision-making are lacking. Standard operating procedures, multicenter benchmarking, transparent reporting, and prospective trials are required before sequencing-guided intervention can enter routine care. Representative metagenomic applications are summarized in **Table 2**.

**Table 2:** Metagenomics research and application in the field of vaginal microecology.

Author and year	Areas of application	Major discoveries and research breakthroughs
(Fredricks et al., 2005)	Pathogenic bacteria detection	The discovery of several novel pathogens associated with bacterial vaginosis (BV).
(Ravel et al., 2011)	Analysis of vaginal microbial diversity	Five primary types of vaginal microbial communities have been identified in healthy women.
(Anahtar et al., 2015)	Prediction of microbial function	Characterizing the metabolic functions of vaginal microbial communities.
(Petrova et al., 2015)	Evaluation of the effectiveness of microecological interventions	This study aims to evaluate the impact of probiotic intervention on the structure of the vaginal microbial community.
(Cardona et al., 2016)	Metabolic interactions between microorganisms	The exchange and transformation of metabolites among various microorganisms facilitate the establishment of either reciprocal or competitive relationships. This metabolic network is crucial for maintaining the stability of microecosystems.
(Gosmann et al., 2017)	Host-microbe interaction studies	The investigation of the mechanisms underlying the interactions between vaginal microorganisms and the host immune system.
(Abubucker et al., 2012; Mallick et al., 2019)	Microbial function prediction: metabolic network-based prediction	By constructing metabolic network models of microbial communities, the metabolic interactions among microorganisms in the communities can be predicted, and the overall functions of the communities can be better understood.
(Yang et al., 2020; Kieft et al., 2020)	Microbial function prediction: sequence-based prediction	The potential metabolic functions of microbial communities can be predicted using 16S rRNA gene sequencing data combined with bioinformatics methods.
(Wang et al., 2022)	Novel functional genes	By analyzing microbial genomes, new functional genes related to vaginal health can be identified, providing new targets for microecological interventions.
(Leon-Gomez and Romero, 2024; Hasan et al., 2024)	Individualized microecological interventions	Through shotgun metagenomics analysis, a personalized micro-ecological intervention program can be developed for each patient to improve the therapeutic efficacy.

## 4 Future directions

Microbiota-directed interventions offer a biologically plausible approach to vaginal dysbiosis, but the maturity of evidence differs substantially by modality. Probiotic and antibiotic-combination studies provide some human data, whereas synbiotics, engineered hydrogels, nanomaterials, VMT, and sequencing-guided treatment remain less established or experimental. Future claims should be calibrated to the level of evidence and supported by strain- or product-specific data.

Future research should prioritize: (a) adequately powered multicenter randomized trials with prespecified sample-size calculations, intention-to-treat analyses, standardized diagnostic and recurrence endpoints, and sufficient follow-up; (b) strain-level functional validation and transparent reporting of dose, viability, route, and manufacturing quality; (c) staged safety and efficacy testing of hydrogels and nanomaterials before clinical claims; (d) rigorous donor screening, traceability, and long-term surveillance for VMT; and (e) harmonized sequencing and multi-omics workflows linked to prospectively validated clinical decision rules. Reproductive outcomes such as HPV clearance, pregnancy, infertility, and ART success should be evaluated as prespecified endpoints rather than inferred from microbiota shifts.

In conclusion, microbiota-directed interventions may complement established therapy for selected vaginal disorders, but the present evidence does not support uniform efficacy across products or indications. The strongest next step is not broader commercial adoption but rigorous comparison of defined interventions using standardized clinical, microbiological, safety, and patient-reported outcomes.
